# Lexical Diversity, Lexical Sophistication, and Predictability for Speech in Multiple Listening Conditions

**DOI:** 10.3389/fpsyg.2021.661415

**Published:** 2021-06-18

**Authors:** Melissa M. Baese-Berk, Shiloh Drake, Kurtis Foster, Dae-yong Lee, Cecelia Staggs, Jonathan M. Wright

**Affiliations:** Speech Perception and Production Lab, Department of Linguistics, University of Oregon, Eugene, OR, United States

**Keywords:** lexical sophistication, lexical diversity, non-native speech, speech in noise, adverse listening conditions

## Abstract

When talkers anticipate that a listener may have difficulty understanding their speech, they adopt a speaking style typically described as “clear speech.” This speaking style includes a variety of acoustic modifications and has perceptual benefits for listeners. In the present study, we examine whether clear speaking styles also include modulation of lexical items selected and produced during naturalistic conversations. Our results demonstrate that talkers do, indeed, modulate their lexical selection, as measured by a variety of lexical diversity and lexical sophistication indices. Further, the results demonstrate that clear speech is not a monolithic construct. Talkers modulate their speech differently depending on the communication situation. We suggest that clear speech should be conceptualized as a set of speaking styles, in which talkers take the listener and communication situation into consideration.

## Introduction

When communicating in natural situations, talkers modulate their speech for their audience (e.g., Clark et al., 1983). Modulation can take many forms, including choosing appropriate lexical items for the audience, modulating syntactic structure, and modifying acoustic properties (Clark and Carlson, 1981; Clark and Murphy, 1982; Arnold et al., 2012). This type of modulation typically happens without explicit instruction or feedback and is robust across talker populations and contexts (Beckford Wassink et al., 2007; Androutsopoulos, 2014; Ferreira, 2019).

The most famous example of this type modulation is child- or infant-directed speech (IDS), a speaking style used, as the name suggests, when communicating with infants or children (Snow, 1977; Stern et al., 1982; Fernald and Simon, 1984). While individual talkers may differ in their exact implementation of IDS, common properties of this speaking style include higher average pitch, a broader pitch range, and shorter utterance durations. Infant-directed speech is not universal (e.g., Pye, 1986; Ingram, 1995); however, it is widely used in many cultures, without explicit instruction (e.g., Grieser and Kuhl, 1988; Fernald et al., 1989; Kuhl et al., 1997). Infant-directed speech also changes in both syntactic and lexical complexity as the infant grows older, presumably in response to increases in infants' receptive abilities as well as their ability to communicate with adult interlocutors (Genovese et al., 2020). Even children can produce IDS in situationally appropriate ways (Dunn and Kendrick, 1982; Warren-Leubecker and Bohannon, 1983; Weppelman et al., 2003), suggesting that the ability to modulate our speech for our audience develops rather quickly and is robust. However, even when speaking to adult listeners, talkers modulate their speech in a variety of ways. For example, a wide range of speaking styles have often been included under the umbrella of “clear speech.” Clear speech is typically defined as a listener-oriented speaking style, characterized primarily by a variety of acoustic modifications. However, recent work has suggested that clear speech differs as a function of the intended audience or communication style (Hazan and Baker, 2011; Hazan et al., 2012) and that clear speech produced in naturalistic communication scenarios differs from clear speech elicited in a laboratory in more artificial communication scenarios (Moon and Lindblom, 1994; Scarborough et al., 2007; Hazan and Baker, 2011; Scarborough and Zellou, 2013).

The bulk of work on clear speech has focused on acoustic modifications of the speech signal, which are thought to make the signal easier for the listener to understand. However, other lines of research have demonstrated that there are multiple other factors that impact how easy it is for listeners to understand the speech they are exposed to. For example, semantic predictability impacts how accurately listeners perceive speech in a variety of challenging listening situations, including speech in noise (Signoret et al., 2018), non-native speech (Baese-Berk et al., 2021), hearing-impaired listeners (Holmes et al., 2018), and cochlear implant users (Winn, 2016).

Indeed, predictability is crucially important for speech understanding and communication in general (see Kutas et al., 2011 for a review). For example, many studies have suggested that listeners use prediction to determine when a speaker is likely to complete their turn so that they can begin the next conversational turn as a speaker (Schegloff et al., 1974). On even shorter time scales, listeners make eye-movements toward relevant targets before they are produced if the target is syntactically or semantically predictable (e.g., Altmann and Kamide, 1999, 2007). Predictability, in various forms, has also been shown to impact language processing. Less predictable words are read more slowly than their more predictable counterparts (Ehrlich and Rayner, 1981; Levy, 2008), and predictability of lexical items is evident in event related potentials (ERPs) to unpredictable lexical items (e.g., N400 responses to semantically less predictable nouns, Kutas and Hillyard, 1980).

In the current paper, we do not directly investigate predictability per se. Instead, we examine lexical factors that could affect the predictability of the speech that listeners hear and could impact the ease of understanding speech. Specifically, we examine speech produced in naturalistic communication scenarios across a variety of contexts known to elicit a clear speech style. We ask whether, in addition to acoustic modifications previously reported, speakers modulate the lexical content of their speech—including a variety of measures of lexical diversity and lexical sophistication. We ask whether these measures differ both when (1) comparing scenarios that naturally elicit clear speech to those that do not elicit such a style and (2) comparing within distinct communication situations that may each elicit clear speech, but differ in their specific challenges for the talker and listener (e.g., speech to a non-native talker vs. to someone hearing the speech through noise).

Below, we briefly review the previous literature on modifications found in clear speech, measures of lexical diversity, and measures of lexical sophistication before turning our attention to the current study.

## Related Work

### Communication in Adverse Listening Situations/Clear Speech

As described above, clear speech is a speaking style adopted by speakers, usually in situations where they anticipate that their listener may have trouble understanding their speech. Substantial previous work has examined the acoustic properties of clear speech. Typical modifications include slower speaking rates, higher average intensity, greater fundamental frequency range, and larger vowel spaces compared to plain or conversational speech (Picheny et al., 1986; Krause and Braida, 2004; Smith, 2007; Maniwa et al., 2009).

Importantly, these modifications result in a benefit for the listener. That is, listeners are able to more accurately transcribe speech (i.e., intelligibility) when the speech is produced in a clear speaking style (Bradlow and Bent, 2002; Krause and Braida, 2002; Maniwa et al., 2008; Hazan and Baker, 2011). These benefits emerge for a variety of listener populations including normal-hearing listeners (Krause and Braida, 2002; Liu and Zeng, 2006; Hazan et al., 2018), hearing-impaired listeners (Picheny et al., 1985; Ferguson and Kewley-Port, 2002), listeners with cochlear implants (Liu et al., 2004), non-native listeners (Bradlow and Bent, 2002), and for speech-in-noise in a variety of populations (Payton et al., 1994; Bradlow and Alexander, 2007; Calandruccio et al., 2020).

Primarily, the studies cited above elicited speech in the laboratory using instructions to produce speech for a hypothetical listener who may have challenges understanding the speech. Some previous work has elicited clear speech with naturalistic methods (Moon and Lindblom, 1994; Scarborough et al., 2007; Hazan and Baker, 2011; Scarborough and Zellou, 2013). In these situations, talkers typically do not receive instructions to modify their speech or to speak clearly. Instead, they are placed in communication situations where their speech will be harder for their listener to understand. There have been some differences reported between these two elicitation types with some showing more hyperarticulation in speech elicited in naturalistic conditions and others showing more hyperarticulation for speech elicited with a hypothetical listener. Importantly, compared to plain speech, both types of elicitation methods result in acoustic modifications and perceptual benefits (see e.g., Hazan and Baker, 2011; Hazan et al., 2015; Lee and Baese-Berk, 2020).

While these previous findings have demonstrated that this listener-oriented speaking style tends to result in both acoustic modifications by the talker and perceptual benefits for listeners, much less attention has been paid to other properties of the language produced by speakers in these situations, especially in clear speech elicited in naturalistic situations. That is, one could imagine that when in a naturalistic environment where communicative success is imperative, talkers may modify their speech in multiple ways, including lexical, syntactic, or pragmatic selection. These modifications could result in even greater ease for listeners. This type of investigation is critically important because some previous work has demonstrated that intelligibility benefits for listeners are not necessarily reflected in acoustic modifications of clear speech (e.g., Lee and Baese-Berk, 2020). That is, in some cases listeners understand speech that was elicited in naturalistic scenarios that often result in “clear speech” better than speech elicited as “plain speech,” but investigations for acoustic correlates that may be driving these results have not shown significant differences between the two speaking styles (e.g., no significant differences in speaking rate, F0, intensity, etc.). Therefore, it is possible that other, non-acoustic, properties of the signal are impacting ease of understanding for listeners.

Further, most previous studies of clear speech have examined the speaking style as a monolithic construct, and have not directly investigated cases in which the specific properties of clear speech might shift as a function of the audience and the needs of the audience. As a counterexample, Hazan et al. (2012) demonstrated that acoustic properties of clear speech differ as a function of communicative barrier (i.e., vocoded speech vs. speech presented in multi-talker babble). For example, speaking rate and fundamental frequency differ across the two conditions—though both are distinct from plain speech. Also, preliminary work from our lab (Wright and Baese-Berk, 2020) suggests that lexical and syntactic information may shift as a function of the needs of the audience. Using only lexical and syntactic information from the talker's speech in transcriptions of conversations from the LUCID corpus, which included three clear speech eliciting conditions and one plain speech eliciting condition, we found that natural language processing classifiers perform significantly above chance when predicting the listening condition of the audience based solely on the talker's speech. This suggests that there are some non-acoustic properties of the speech that are differentiated among the various clear speech eliciting conditions. However, the factors differentiating lexical and syntactic properties that allowed the classifiers to perform well were not clear.

There is a broad body of work on how interlocutors refer to objects in the world in conversation (see Arnold, 2008 for a review). When speaking, we have the choice of many different ways to refer to the same referent in the world (e.g., *the cat, it, the striped one*), and the method of reference we select seems to depend on many factors. Among these factors are whether the information being referred to is new or given (i.e., previously referred to in discourse), what a speaker knows about a listener's familiarity with the topic, other information that the speaker infers about the listener (e.g., proficiency in the language of discourse), and ease of retrieval for the speaker. Thus, it seems that the notion of what constitutes “clear speech” can be even further subdivided.

Therefore, here, we investigate one specific aspect that could be modified by talkers during elicitation of clear speech in naturalistic conversations: lexical selection. Below, we briefly describe the two families of measurements used in our analyses: lexical diversity and lexical sophistication. Both families of measures are used widely in assessment of second language writing, among other fields. We believe that they are appropriate for the present study because they provide us with a series of measures capable of directly assessing lexical complexity, which may impact how listeners perceive speech and/or how speakers modify their speech for listeners.

### Lexical Diversity

Broadly speaking, lexical diversity is the range of different words used in a text or conversation. A greater range is equivalent to higher diversity. Lexical diversity is used in a variety of assessment tools including as a measure of proficiency in a second language (Engber, 1995; Cumming et al., 2005), vocabulary knowledge (Zareva et al., 2005; Yu, 2010), and even as a marker of onset of neurodegenerative diseases like Alzheimer's disease (Garrard et al., 2005; van Velzen and Garrard, 2008) or in mild cases of aphasia (Cunningham and Haley, 2020). Measures of lexical diversity are important for many reasons. While more diverse texts or speech samples may be indicative of greater proficiency for the speaker or writer, they may also be more challenging for a reader or listener to understand. That is, samples with greater diversity, may also include less repetition, more switches among topics, and use of multiple lexical items to refer to the same concept. Each of these factors could make it *more* challenging for a listener to understand what is being said. Therefore, we may expect *lower* lexical diversity values in clear speech situations than in plain speech situations.

Historically, lexical diversity has been indexed via the type-token ratio (Johnson, 1944; Templin, 1957), in which the total number of unique words (i.e., types) is divided by the total number of words (i.e., tokens). The closer this ratio is to 1, the greater lexical diversity in the sample. However, indices like type-token ratio are often sensitive to length of language sample: longer texts often have disproportionately lower type-token ratios than shorter texts, and this value may not be indicative of lexical diversity more broadly. Further, some measures of lexical diversity (including type-token ratio), make assumptions about textual homogeneity. That is, some measures of lexical diversity fail to recognize that talkers may vary diversity levels in different points of conversation or a text for some specific purpose. For instance, there are particular circumstances in which language that is less lexically diverse is employed as a rhetorical strategy, therefore, indices have been developed that control for the intentional use and variety of particular structures. This serves to ensure that the measure does not treat a single structure or pattern as representative of the text as a whole (see McCarthy and Jarvis, 2010 for a summary of these issues).

In the present study, we present results from the typical type-token ratio analyses. However, given the considerations above, we also report three additional measures, which may provide a more complete understanding of lexical diversity within our sample. First, we report the moving average type-token ratio (MATTR; Covington and McFall, 2010), which uses a 50-word window to continuously calculate type-token ratio throughout a sample. Second, we report the hypergeometric distribution (HD-D; McCarthy and Jarvis, 2007). This represents the probability of drawing a number of tokens with some specific type from a sample of a specific size. Finally, we report a version of the “measure of textual lexical diversity” (MTLD; McCarthy, 2005; McCarthy and Jarvis, 2010). While we refer the reader to previous work for specific descriptions of this index, the measure roughly corresponds to the average length in words that the sample stays at a specific type token ratio.

Taken together, we believe these indices will allow us to better understand the lexical diversity of the samples in the current study. By comparing how these indices differ across a number of conditions that induce clear speech, we will be able to better understand how clear speech may vary across scenarios.

### Lexical Sophistication

Lexical sophistication is often simply described as the number of “unusual” words in a sample. As is the case for lexical diversity, a number of constructs can be used for characterizing lexical sophistication, depending on the goals of the researcher (Eguchi and Kyle, 2020). Lexical sophistication is frequently used as an indicator of language proficiency in second language assessments of speaking and writing (Laufer and Nation, 1995; Kyle and Crossley, 2015; McNamara et al., 2015). However, we believe that it could be a tool to characterize the relative lexical complexity of clear speech, as in the current study.

Here, we specifically assess four measures of lexical sophistication (see Crossley et al., 2012), all of which investigate the relative frequency of a word or sets of words. First, we report the lexical frequency for words within our speech samples. This frequency is calculated using a reference corpus. The reference corpus should, ideally, match the properties of the speech sample, given that relative frequency of a word, for example, may differ across language variety or modality (i.e., spoken vs. written). We discuss this issue in more detail below. Second, we report the range, or the number of speech samples in a particular corpus in which a word occurs. Third, we report two measures of bigram frequency in a sample: the mean frequency for bigrams (i.e., pairs of words) and the proportion of bigrams in the sample that are within the most frequent 25,000 bigrams in the corpus. Finally, we report the same two measures for trigrams (sets of three consecutive words).

We interpret measures of lexical sophistication as being indicative of lexical complexity within our clear speech and plain speech samples. We predict that, if talkers modify their lexical complexity for their audience, they will use higher frequency words and higher frequency collocations (i.e., bigrams and trigrams) when producing clear speech than plain speech.

### Current Study

In the current study we examine talker speech modulations across naturalistic scenarios in the London UCL Clear Speech in Interaction Corpus (LUCID; Baker and Hazan, 2011; Hazan and Baker, 2011). The LUCID corpus includes naturalistic conversations in a variety of conditions designed to elicit clear speech, as well as a “no-barrier” condition that elicit naturalistic conversation between native English speakers. The clear speech conditions include speech in noise, a simulation of speech through a cochlear implant (i.e., vocoded speech), and conversations between individuals who do not share a language background (i.e., native English speakers and non-native English speakers). Previous studies have used this dataset to demonstrate that talkers make acoustic modifications of their speech in clear-speech situations (Hazan and Baker, 2011) and that speech in clear-speech situations is more easily understood than speech in plain-speech situations (Hazan and Baker, 2011; Lee and Baese-Berk, 2020). To determine how speakers might modulate other aspects of their speech, we use measures of lexical diversity and lexical sophistication to directly investigate how talkers modulate lexical selection across clear-speech eliciting conditions and plain-speech eliciting conditions.

Specifically, we compare lexical selection in clear-speech eliciting conditions to a condition not designed to elicit clear speech. As previous studies have shown robust acoustic differences between the two broad speaking styles, we ask whether lexical diversity and lexical sophistication also differ between these styles.

We also compare clear-speech eliciting conditions with L1 listeners to speech directed to L2 listeners. We ask whether speech to L2 listeners without an additional barrier to communication differs from speech to L1 listeners in communicatively challenging situations (speech in noise; a simulation of speech through a cochlear implant). Most work on clear speech refers to the clear-speech speaking style as “listener-oriented,” and groups clear-speech eliciting conditions together under the same umbrella. However, here, we ask whether clear-speech eliciting conditions are actually the same and whether talkers are orienting their speech toward some generic listener who may have difficulty understanding them or whether this modulation is more dynamic in nature. While clear-speech eliciting conditions may share some properties, they may also differ in ways that are important to understand if we are to fully account for how talkers modulate their speech for their audience.

Finally, we compare clear-speech eliciting conditions with L1 speakers directly to each other, asking whether measures of lexical diversity and lexical sophistication reveal differences in lexical selection in speech to L1 listeners as a function of the challenging listening situation, which expands on previous work that has demonstrated that there are acoustic features that differ as a function of the communication challenge faced (Hazan et al., 2012).

## Methods

In this study, we analyze data previously collected for the LUCID corpus. Below, we briefly describe the participants and task before describing more detail the specific stimuli we analyzed in the present paper, the measures we extracted, and the analyses conducted. For more in depth descriptions of the participants and task, we direct the reader to Baker and Hazan (2011). Further, all sound files and transcripts analyzed in this project are publicly available via SpeechBox (Bradlow^1^).

### Participants

Participants in this task were 40 native, monolingual speakers of southern British English, between 18 and 29 years of age. 20 participants identified as female, and 20 participants identified as male. Participants did not self-identify as having a history of speech or hearing disorder and all participants passed a basic hearing screening.

### Task

Each participant in the LUCID Corpus completed a set of Diapix tasks (Van Engen et al., 2010). Participants in this task completed a “spot-the-differences” task. Each participant is presented with a different hand-drawn picture that is very similar to their partner's picture but contains several key differences. These differences can include missing items (e.g., a sign being present in one picture but absent in the other) or differences in objects or actions (e.g., a girl sitting on a beach ball in one picture but playing with the beach ball in the other picture). Differences in missing items are equally distributed between picture pairs. Participants are asked to collaborate with their partner to find 10 differences between their pictures without seeing their partner's picture (see Baker and Hazan, 2011 for pictures used in the Diapix tasks). This task requires both partners to contribute to solving the task, resulting in a different balance of speech across talkers than tasks like the Map Task (Anderson et al., 1991), which has a set giver-receiver structure. The range of items in a Diapix picture allows the experimenter to more closely limit the lexical items that will be discussed in the picture than a free-ranging conversation, and, at the same time, the specific structure of the pictures described in the LUCID corpus (i.e., DiapixUK) requires participants to use a variety of linguistic structures to accurately complete the task.

The LUCID corpus includes talkers describing one of three different types of scenes: beach, farm, or shop. Each participant completed each scene with a different partner or communication situation. During session 1, all talkers completed the task in quiet listening conditions. During session 2, the target talkers spoke to partners who heard vocoded speech (i.e., cochlear implant simulations). During session 3, talkers spoke with a partner who either heard the speech in multi-talker babble (i.e., noise) or a partner who is a native speaker of a non-English language and is a low-proficiency English speaker. Therefore, speech was produced in one of four conditions analyzed below. We adopt the terminology used by Hazan and colleagues in their work to refer to these conditions: no-barrier (i.e., conversational/plain speech), vocoded (i.e., cochlear implant simulation), babble (i.e., speech-in-noise), and L2 (i.e., speech with a communication partner who is a non-native speaker). No talkers produced speech in all conditions; however, all talkers produced speech in three of the four conditions. Further, the order of the pictures was counterbalanced across talkers, thus any effects below cannot be accounted for solely by picture content or picture order. By examining speech from the same set of talkers, we also hope to roughly control for individual differences in how talkers modulate their speech for an audience.

### Stimuli

The LUCID corpus contains sound files for each conversation and each conversation is orthographically transcribed in time-aligned TextGrids. For this project, we used the Praat TextGrids (Boersma and Weenink, 2021) associated with each sound file to extract the speech from the target talker for each conversation. Here, we define the target talker as the talker who does not experience the communication barrier (i.e., not hearing speech in babble or through a vocoder). The transcriptions were cleaned to prepare them for tokenization (i.e., dividing the transcript into individual words) and lemmatization (i.e., modifying the words into uninflected lexical items) using the Tool for the Automatic Analysis of Lexical Diversity (TAALED) and Tool for the Automatic Analysis of Lexical Sophistication (TAALES) interfaces (described below). All filled and unfilled pauses, as well as other vocal noises (i.e., laughter) were removed from the transcriptions.

### Measurements

Using the transcripts described above, we extracted a series of lexical diversity and lexical sophistication measures. For the lexical diversity measures, we used the TAALED (Kyle et al., 2021). This tool allows for extraction of typical measures of lexical diversity (e.g., type-token ratio), but also a variety of more complex measures of diversity (e.g., MTLD). For the lexical sophistication measures, we used the TAALES (Kyle and Crossley, 2015; Kyle et al., 2018).

Tool for the Automatic Analysis of Lexical Diversity calculates lexical diversity within a single spoken or written text, and thus does not require a reference corpus. Tool for the Automatic Analysis of Lexical Sophistication, on the other hand, calculates frequency information and other measures in reference to larger corpora, and thus requires a reference corpus. Because our speakers in this study were all native speakers of southern British English, we used the British National Corpus (BNC Consortium, 2007) as our reference corpus. Specifically, we used the spoken-language sections of the corpus, since we are examining spoken language, not written language^2^.

### Analyses

We conducted linear mixed models for each measurement of interest. For each measurement, the measurement (e.g., type-token ratio) was the dependent variable. Condition was the fixed factor. We Helmert coded condition to make the following comparisons: (1) no-barrier condition vs. barrier conditions (L2, vocoded, and babble); (2) L2 vs. other barrier conditions (i.e., babble and vocoded speech); and (3) babble vs. vocoded speech^3^.

Our reasoning for including these comparisons was as follows: First, we need to understand whether participants modify these factors when producing speech in challenging listening situations in general vs. in an “easy” listening condition. The first comparison answers this question. Second, the three barrier conditions all differ from each other, but the L2 condition differs from the other two conditions in that both of those conditions have a similar listener (i.e., L1 listener). The second comparison allows us to ask whether the language background of the interlocutor corresponds to specific modifications of lexical selection by the talker. Finally, we ask whether the two conditions with an L1 listener in a challenging situation differ from one another through the third comparison.

In all models, we include talker as a random intercept. Inclusion of other random effects (e.g., scene) resulted in overfitting of the models and are thus not included (Barr et al., 2013).

Significance of each factor was calculated using model comparisons where a model without the factor in question was compared to a model including that factor. Tables containing full model results are included in Supplementary Material. Below, we summarize the model comparison results.

## Results

Below, we present analyses for each of the indices we have calculated. First, we present the results for lexical diversity, followed by the results for lexical sophistication. In all cases we investigate all words produced, rather than subsetting to content words or function words. In general, content words show similar patterns to the full set of words. Patterns for function words differ slightly, but we believe that this is largely driven by the fact that function words in general are a smaller set of words which skew these measures. Therefore, below we report the analyses for all words.

### Lexical Diversity

Before examining specific indices, it is useful to note how much speech is produced in each condition. Because it is clear that some lexical diversity measures are sensitive to length of sample, we begin by reporting the average number of tokens in each sample for each condition. This is shown in Table 1 below:

**Table 1:** Average number of words (i.e., tokens) per conversation per condition.

**Condition**	**Average number of tokens**
No-barrier	662.78
L2	1,095.92
Babble	756.75
Vocoded	785.21

It is clear that talkers produce the most speech when communicating with an L2 listener and the least speech when speaking in the “no-barrier” condition. The two other “barrier” conditions (babble and vocoded speech) are intermediate, but are closer to the no-barrier condition than to the L2 condition. This suggests that if we find effects of lexical diversity with indices that are sensitive to sample length (e.g., type-token ratio) these effects may be driven by these rather large differences in text length. We still report these results below because we believe that a picture from all metrics is informative.

#### Type-Token Ratio

All three main effects were significant for the analysis of type-token ratio. The comparison of the no-barrier condition to the other three conditions significantly improved model fit (χ^2^ = 139.8, *p* < 0.0001). The comparison of the L2 condition to the other two barrier conditions (babble and vocoded) also significantly improved model fit (χ^2^ = 60.916, *p* < 0.0001). Finally, the comparison between the babble and vocoded conditions also significantly improved model fit (χ^2^ = 6.15, *p* = 0.013).

**Figure 1:**
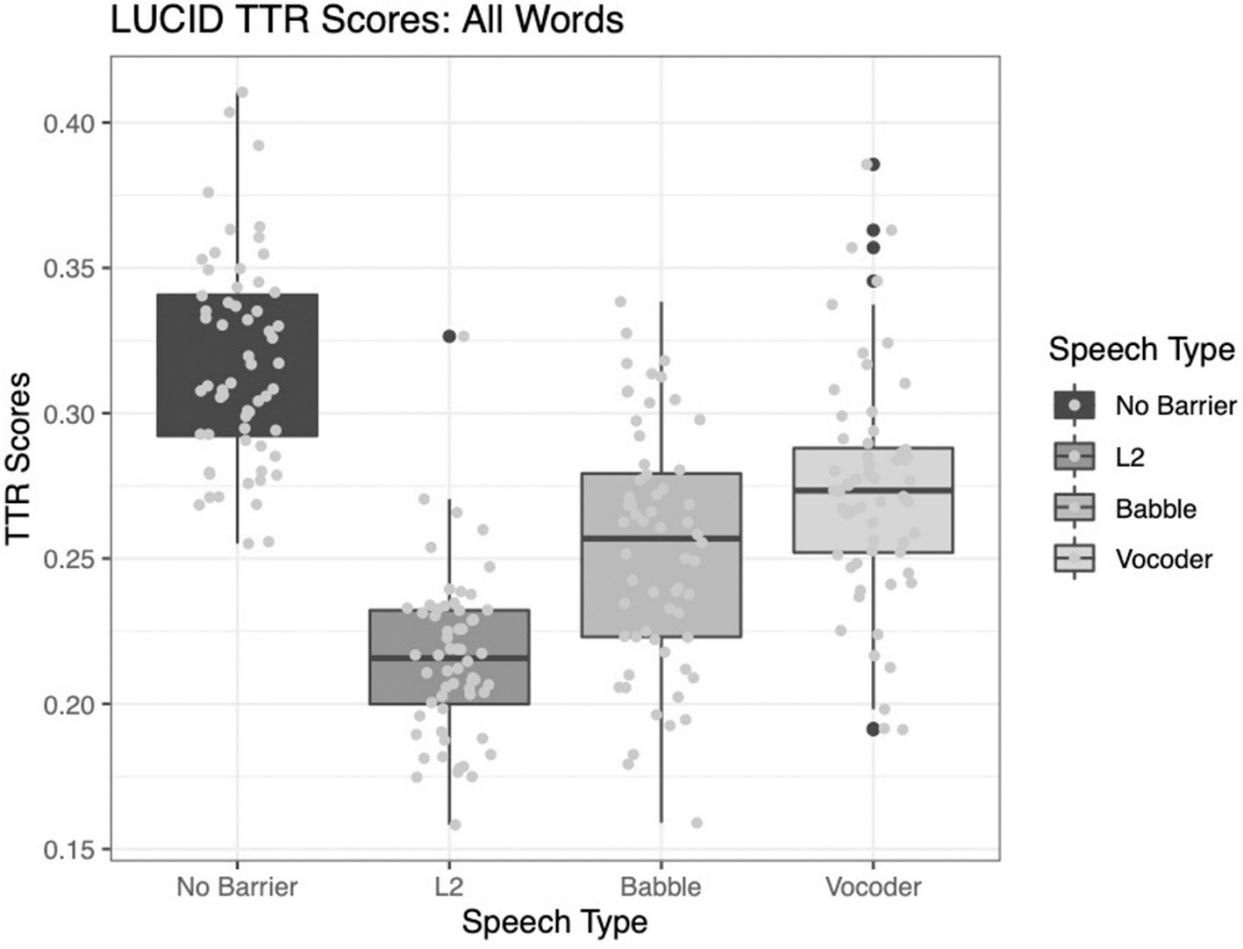
Type-token ratio across four conditions.

Examining Figure 1 below, it is clear that the type-token ratio is highest for the no-barrier condition, compared to the other conditions. Further, the L2 condition demonstrates the lowest type-token ratio, and the other two conditions are intermediate, with the vocoded condition showing a higher type-token ratio than the babble condition. This is in line with our prediction that talkers might use more repetitive speech in the “barrier” conditions than the no-barrier condition. However, this is also in line with previous findings suggesting that type-token ratio may be sensitive to sample length. Therefore, we now turn our attention to more sophisticated measures of lexical diversity.

#### Moving Average Type-Token Ratio

As in the case of type-token ratio, all three main effects were significant for the analysis of the MATTR (calculated over a 50-word window). The comparison of the no-barrier condition to the other three conditions significantly improved model fit (χ^2^ = 149.1, *p* < 0.0001). The comparison of the L2 condition to the other two barrier conditions also significantly improved model fit (χ^2^ = 7.85, *p* = 0.005). Finally, the comparison between the babble and vocoded conditions also significantly improved model fit (χ^2^ = 20.037, *p* < 0.001).

**Figure 2:**
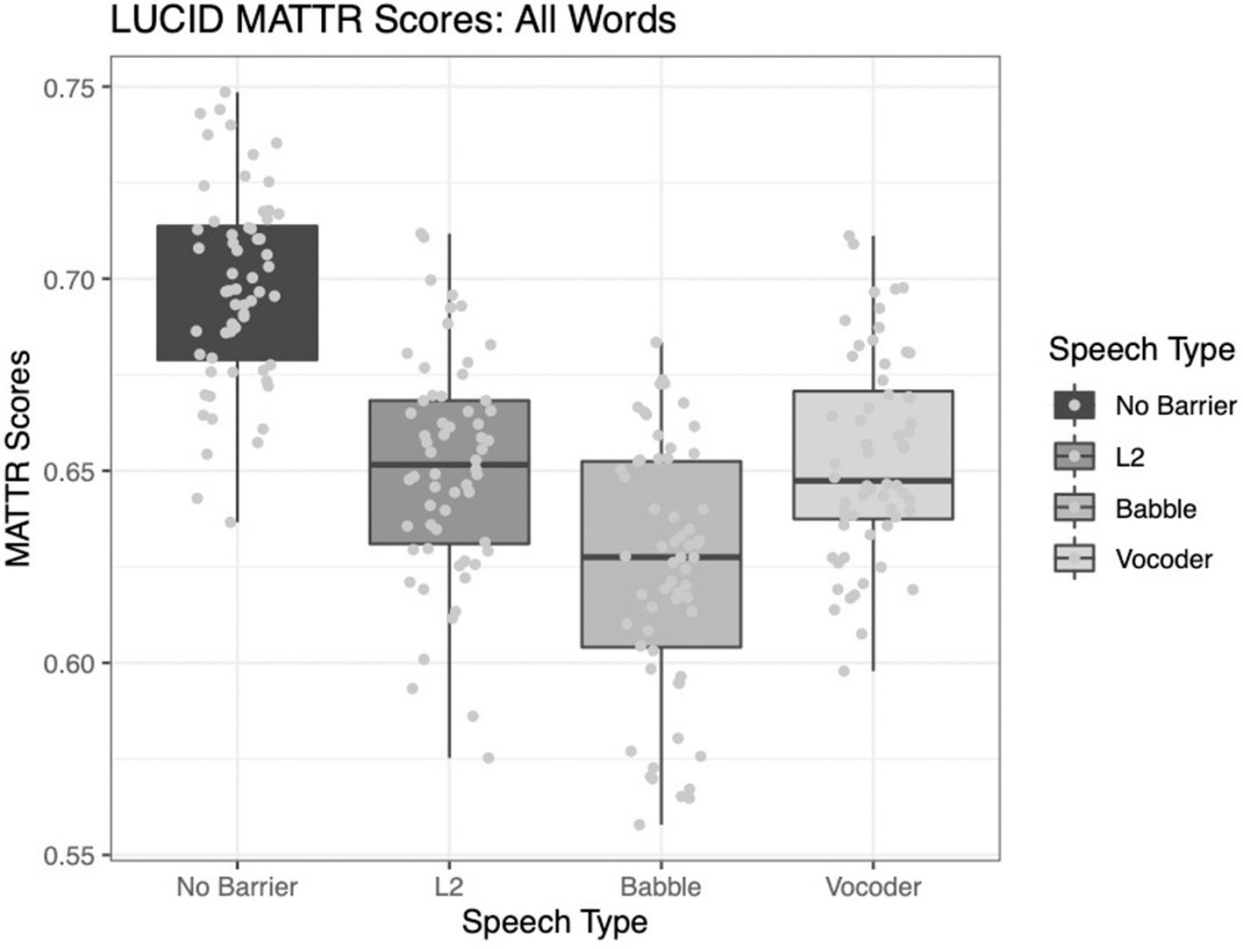
Moving-average type-token ratio calculated over a 50-word window across four conditions.

As demonstrated in Figure 2 below, it is clear these results fall in line with those results for the basic type-token ratio described above.

#### Hypergeometric Distribution

Here, the results differ from the two type-token ratio analyses described above. Two of the main effects significantly improve model fit. The comparison of the no-barrier condition to the other three conditions significantly improved model fit (χ^2^ = 80.207, *p* < 0.0001). Further, the comparison between the babble and vocoded conditions also significantly improved model fit (χ^2^ = 8.9887, *p* = 0.003). However, the comparison of the L2 condition to the other two barrier conditions does not significantly improve model fit (χ^2^ = 0.1698, *p* = 0.6803).

**Figure 3:**
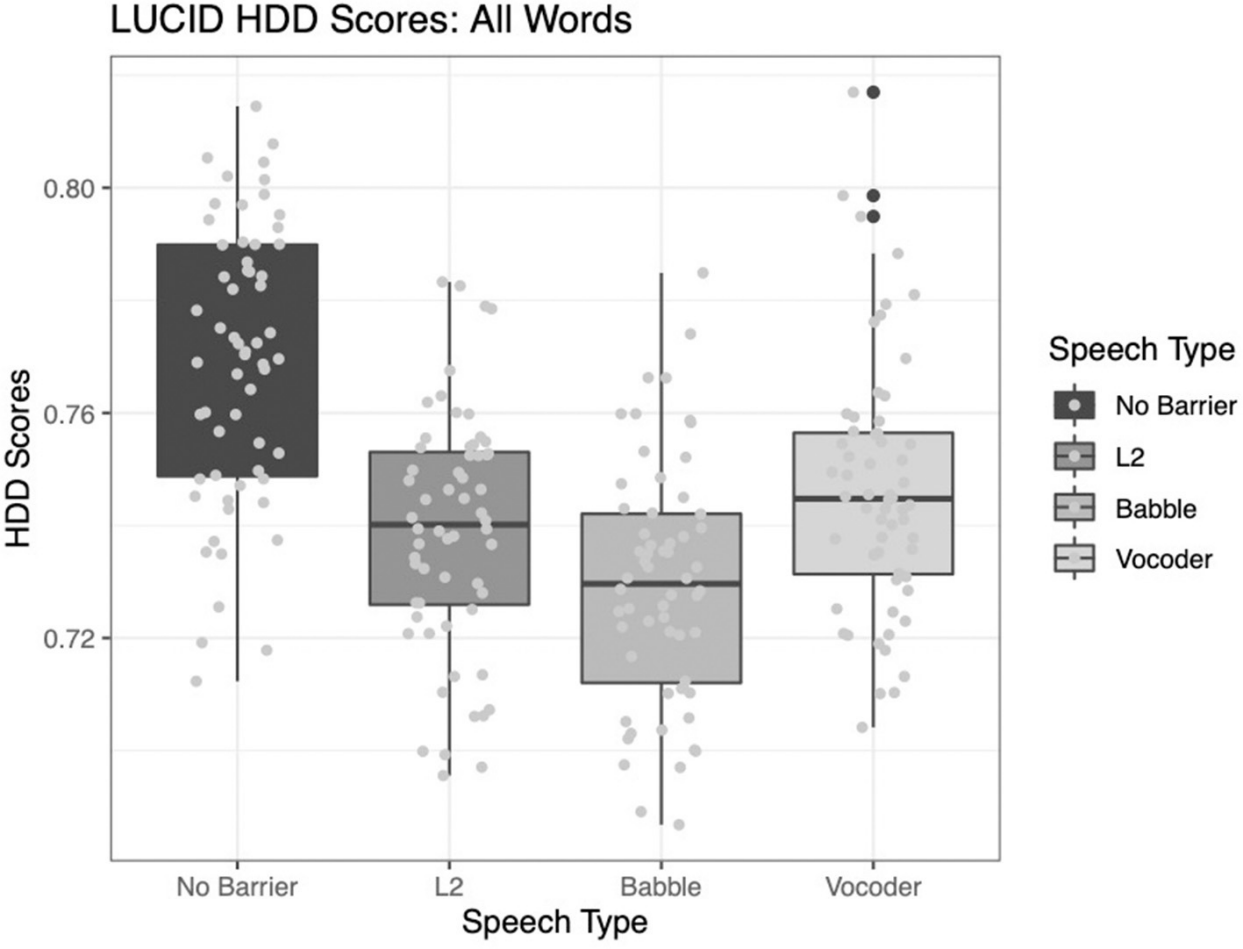
Hypergeometric distribution (from a random sample of 42 tokens); converted to the same scale as type-token ratio across four conditions.

Figure 3 shows the results for this index. Note that HD-D is designed to control for the assumption of homogeneity in the sample, more than for the imbalance in text size, suggesting that when controlling for homogeneity, speech to L2 listeners may be similar in terms of lexical diversity to speech in the other two barrier conditions.

#### Measure of Textual Lexical Diversity

As in the case of the type-token ratio indices reported above, all main effects significantly improve model fit. The comparison of the no-barrier condition to the other three conditions significantly improved model fit (χ^2^ = 119.69, *p* < 0.0001). The comparison of the L2 condition to the other two barrier conditions (babble and vocoded) also significantly improved model fit (χ^2^ = 4.3075, *p* = 0.038). Finally, the comparison between the babble and vocoded conditions also significantly improved model fit (χ^2^ = 8.2303, *p* = 0.004).

**Figure 4:**
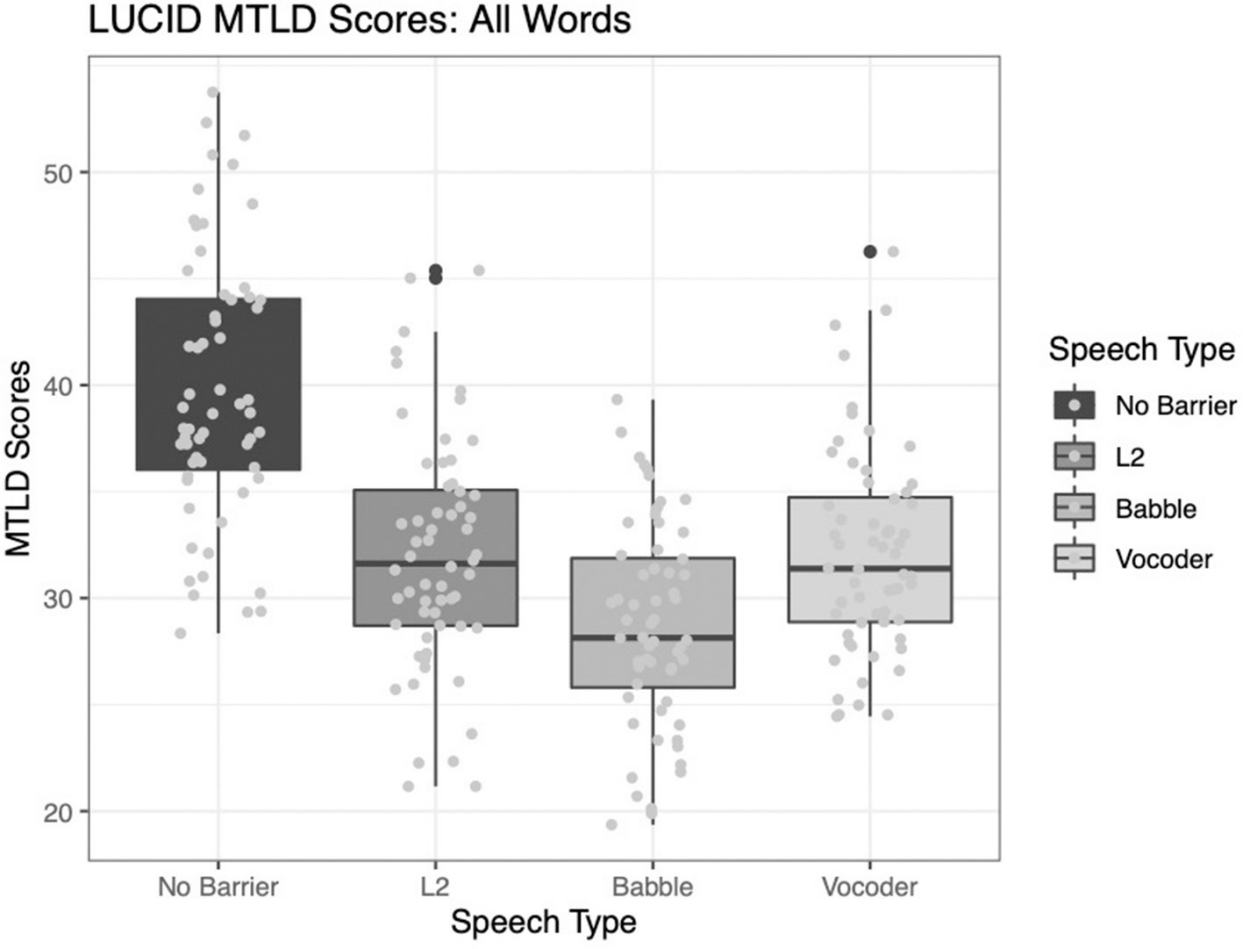
Measure of textual lexical diversity (using a moving average approach, both forward and backward) across four conditions.

Figure 4 depicts the MTLD indices for each condition.

#### Order Effects

One concern with the results here is that participants perform the task multiple times, and thus the order of conditions may impact the results. The order of conditions was fixed across participants such that all participants first completed the no barrier condition followed by the vocoded condition. Half the participants then completed the babble condition and half of the participants completed the L2 condition. Therefore, condition order is conflated with condition type for this study. However, given the results, we believe that order of condition is not a major concern for our study. That is, one might expect that over time participants would repeat words more often (i.e., have lower lexical diversity measures). If this were the case, we would expect that the L2 and babble conditions should have the least lexical diversity. While it is the case that, in general, these conditions have less lexical diversity than the no barrier condition, they do not differ systematically from the vocoder condition. Therefore, we believe it is unlikely that order of conditions alone explains our results. This interpretation is in line with evidence from Baker and Hazan (2011), who demonstrated that these participants did not appear to improve or “learn” across iterations of completing this task.

A second concern is that the order of pictures within a condition may impact performance. Each participant completed three pictures within each condition. However, order of picture was not a significant predictor of model fit for any of the above metrics, and was therefore not included in the final model fit for any metric. This is consistent with evidence suggesting participants do not complete the task more quickly across iterations of the pictures (Lee and Baese-Berk, 2020).

#### Interim Summary

Taken together, these results suggest that there are significant differences in lexical diversity between conditions that are and are not designed to elicit clear speech. The no-barrier condition shows the most lexical diversity, whereas the L2 condition, generally, shows the least diversity. There are some differences across metrics in terms of the relative ranking of diversity values for the babble and vocoded conditions, suggesting that these two conditions may be more similar to one another than to either the no-barrier or L2 conditions.

### Lexical Sophistication

As in the case of the lexical diversity results presented above, we describe each index in turn below.

#### Lexical Frequency

Two of the main effects significantly improved model fit for the analysis of lexical frequency. The comparison of the no-barrier condition to the other three conditions significantly improved model fit (χ^2^ = 66.666, *p* < 0.0001). The comparison of the L2 condition to the other two barrier conditions (babble and vocoded) also significantly improved model fit (χ^2^ = 12.225, *p* = 0.0005). However, the comparison between the babble and vocoded conditions did not significantly improve model fit (χ^2^ = 3.3212, *p* = 0.068).

**Figure 5:**
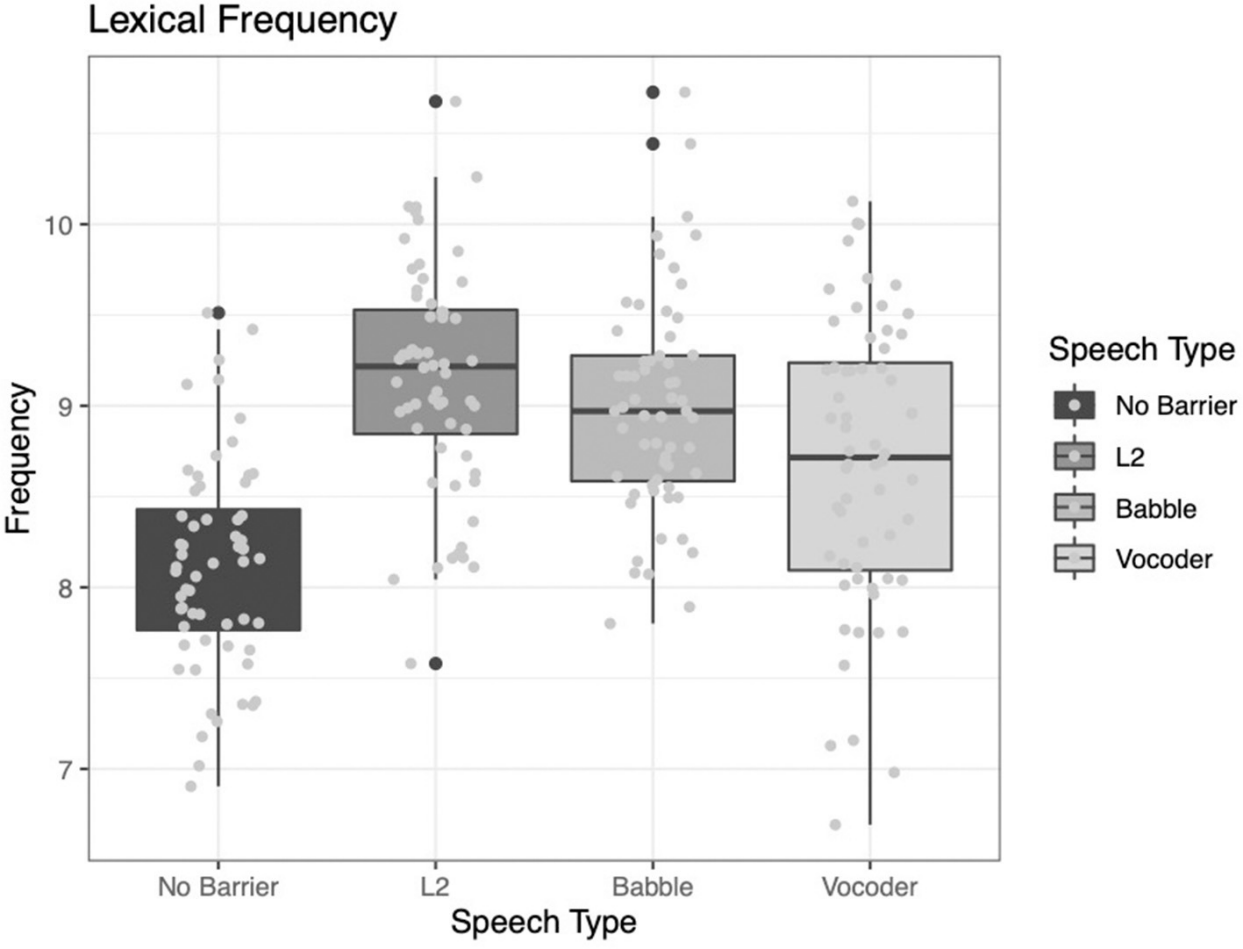
Lexical frequency from the LUCID corpus across four conditions.

Examining Figure 5 below, it is clear that lexical frequency is the lowest for the no-barrier condition and highest for the L2 condition. As in the case of the lexical diversity measures presented above, the other two conditions fall intermediate to these conditions. While numerically the babble condition shows higher frequency than the vocoded condition, this difference was not significant. This result suggests that speakers modify not only the variability in words they produce, but also specifically *which* words they produce. We continue to explore these effects with the indices below.

#### Range

The pattern of results for range is different from any of the previously reported results. Recall that range here refers to the number of samples in the reference corpus (i.e., BNC) that a word appears in. Another way of describing this metric is how “common” the word is. Here, we see that the comparison of the no-barrier condition to the other three conditions *did not* significantly improve model fit (χ^2^ = 0.6595, *p* = 0.4168). This is notable because, thus far, all analyses have suggested significant differences between the conditions designed to elicit clear speech (i.e., barrier conditions) and the condition designed not to elicit clear speech (i.e., no-barrier condition). To further complicate the puzzle, the other two main effects *do* significantly contribute to model fit. The comparison of the L2 condition to the other two barrier conditions (babble and vocoded) significantly improved model fit (χ^2^ = 59.877, *p* < 0.0001). Further, the comparison between the babble and vocoded conditions also significantly improved model fit (χ^2^ = 7.7695, *p* = 0.005).

**Figure 6:**
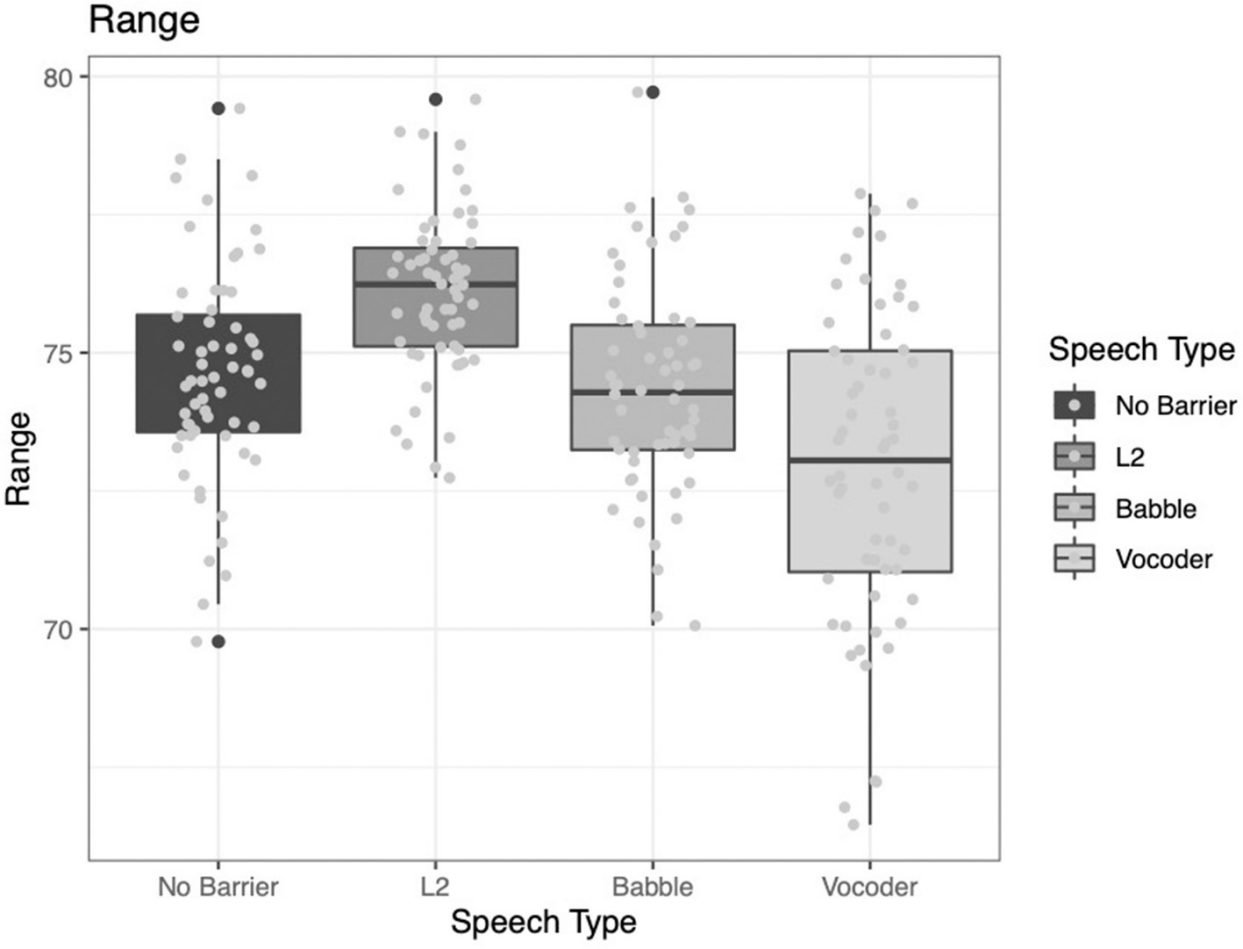
Range of samples from the BNC in which a word from the LUCID corpus was found across four conditions.

Examining Figure 6 below, it becomes clear that the pattern of results is different from the patterns demonstrated for the other indices. While we continue to observe more common words (i.e., a greater range) for the L2 condition, it is not the case that the no-barrier condition follows the typical patterns observed above. Specifically, instead of the no-barrier condition being the lowest value, the vocoded condition is the lowest. It is not immediately clear why this would be the case; however, it is possible that because the vocoded condition is the least familiar to participants they may demonstrate less consistency across indices, compared to the other conditions. That is, the other three conditions are cases that talkers are likely to have at least some familiarity with. Talking to someone in a noisy environment is a common occurrence at a restaurant or party. Speaking with a non-native speaker is also a relatively common occurrence for many talkers in our increasingly globalized society. However, speaking to someone who is perceiving your speech through a vocoder is relatively rare. Even if a person does have experience communicating with someone with a cochlear implant, it is unlikely they would have experience hearing that type of speech as well. Here, all participants are familiarized with how speech sounds when vocoded, which could impact how they modify their speech.

#### Bigram Frequency

For bigram frequency, we see that the comparison of the no-barrier condition to the other three conditions significantly improved model fit (χ^2^ = 26.318, *p* < 0.0001). However, the other two comparisons did not significantly improve model fit (L2 vs. other conditions: χ^2^ = 0.5585, *p* = 0.4549; babble vs. vocoded: χ^2^ = 0.2157, *p* = 0.6423).

**Figure 7:**
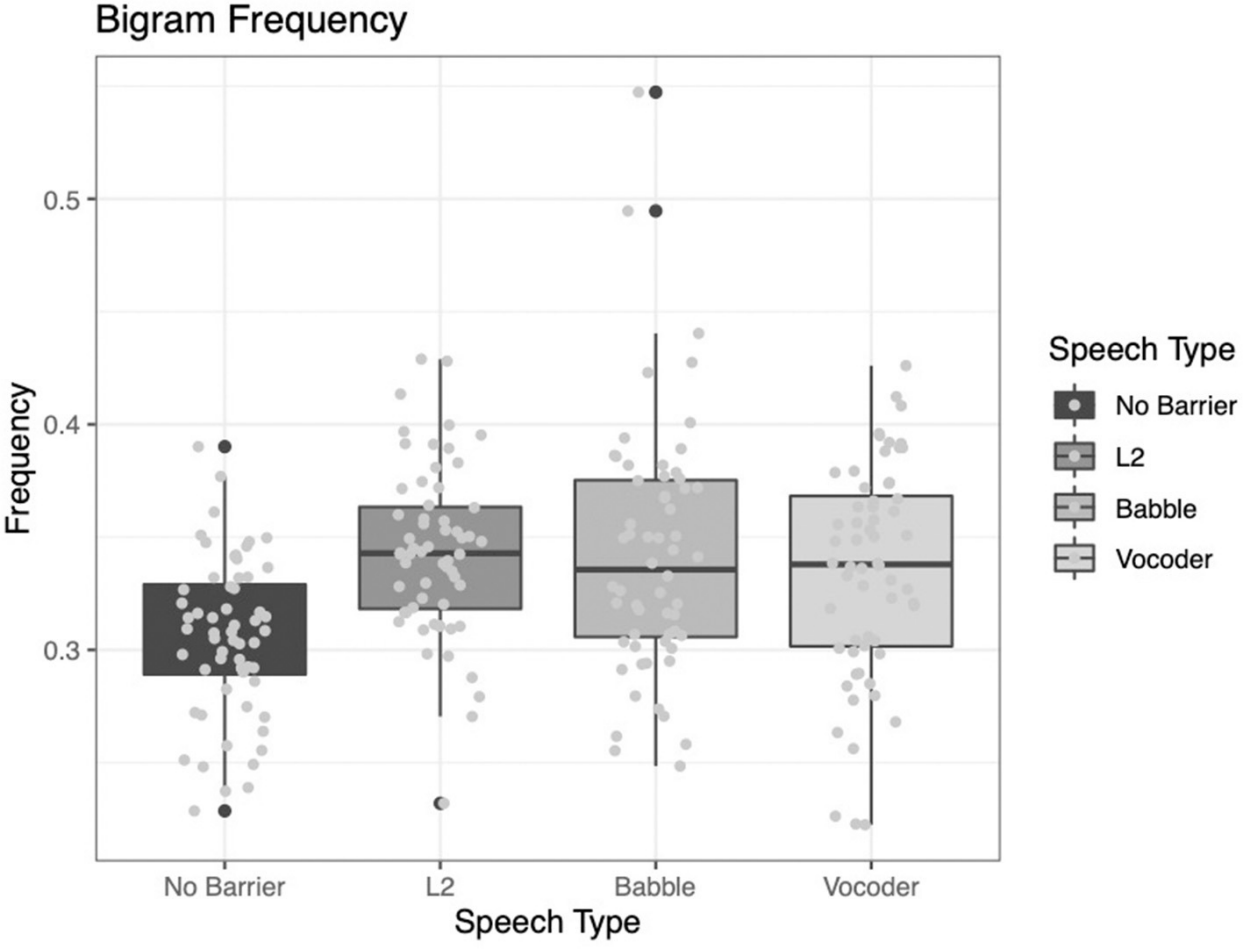
Frequency of bigrams (i.e., pairs of words) from the LUCID corpus across four conditions.

These results, too, diverge from some of the previously reported results. Examining Figure 7 below, we see that while the no-barrier condition shows the lowest bigram frequency, the other three conditions do not differ significantly from one another.

#### Trigram Frequency

The trigram frequency analysis reveals that none of the main effects significantly improve model fit (No-barrier vs. barrier conditions: χ^2^ = 2.5851, *p* = 0.1079; L2 vs. other conditions: χ^2^ = 1.7431, *p* = 0.1867; babble vs. vocoded: χ^2^ = 1.9693, *p* = 0.1605).

**Figure 8:**
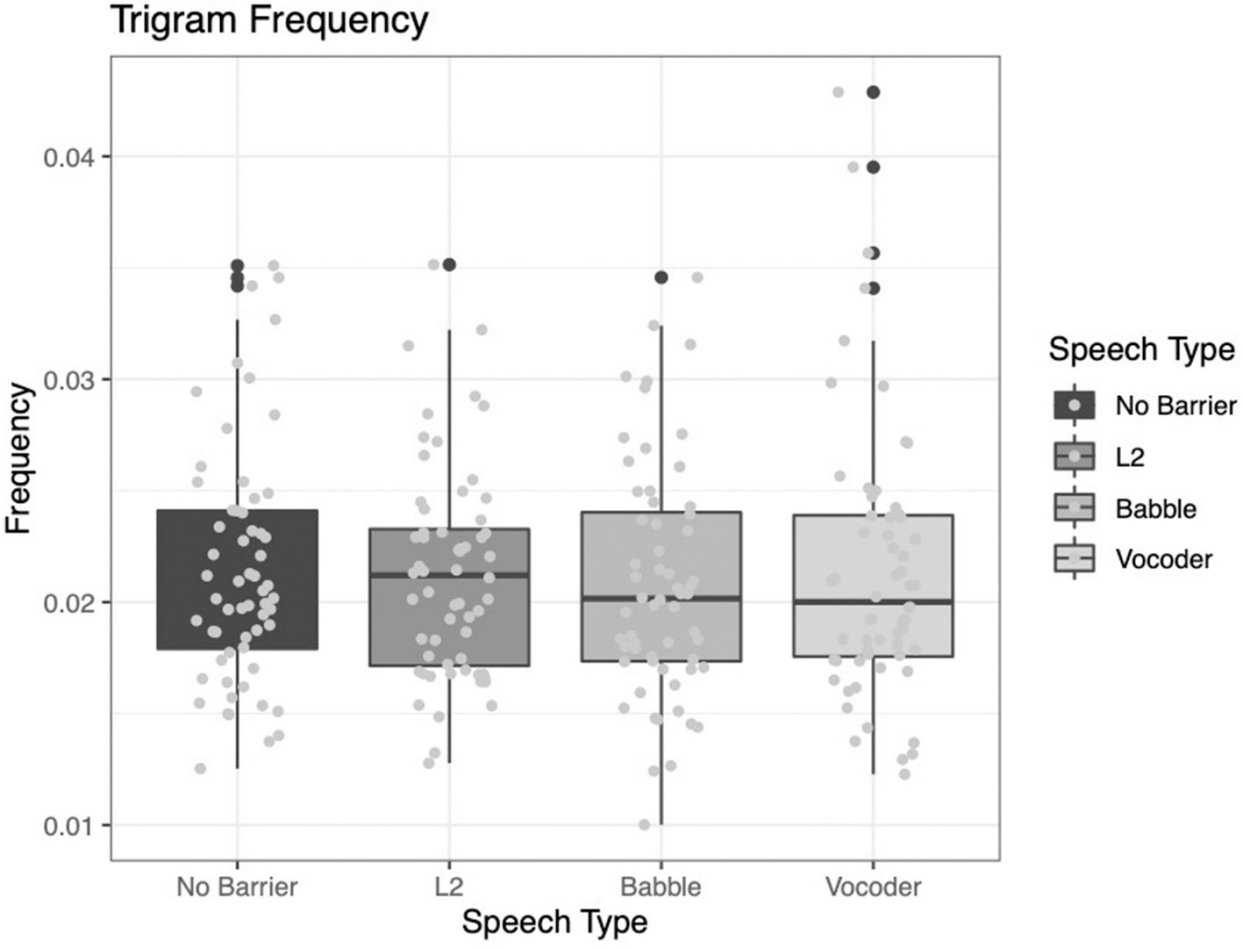
Frequency of trigrams (i.e., sets of three of words) from the LUCID corpus across four conditions.

While numerically the results fit with our previous observations (see Figure 8), because no results are significant, it is difficult to interpret these findings. We are especially cautious not to overinterpret the null results we observe here.

#### Proportion of Bigrams Within the 25,000 Most Frequent Bigrams

Rather than looking at frequency of the two- and three-word collocations in our samples, here we examine the proportion of these collocations that are among the most frequent bigrams (and then trigrams) in the corpus.

As was the case for lexical frequency, two of the main effects significantly improved model fit for the analysis of lexical frequency. The comparison of the no-barrier condition to the other three conditions significantly improved model fit (χ^2^ = 13.932, *p* = 0.0002). The comparison of the L2 condition to the other two barrier conditions (babble and vocoded) also significantly improved model fit (χ^2^ = 57.597, *p* < 0.0001). However, the comparison between the babble and vocoded conditions did not significantly improve model fit (χ^2^ = 2.9483, *p* = 0.086).

**Figure 9:**
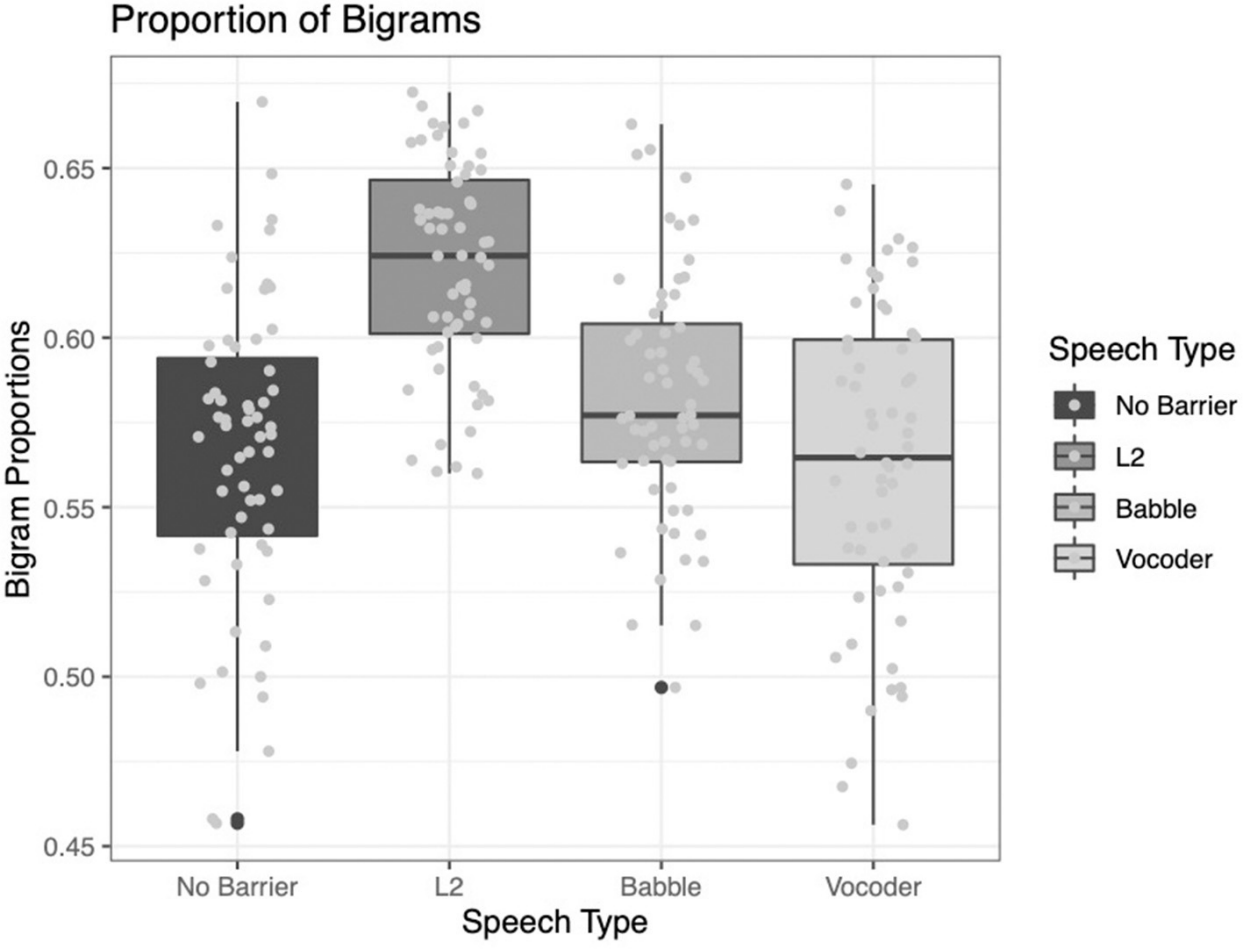
Proportion of bigrams from the LUCID found among the 25,000 most common in the BNC.

Examining Figure 9 below, it is clear that this index follows the pattern of many of the indices above. The no-barrier condition reports the lowest proportion of bigrams among the 25,000 most frequent and the L2 condition reports the highest proportion, with the other conditions lying intermediate between the two.

#### Proportion of Trigrams Within the 25,000 Most Frequent Trigrams

For this index, the only factor that emerged as significantly contributing to model fit was the comparison of the L2 condition to the other barrier conditions (χ^2^ = 48.845, *p* < 0.0001). The other two comparisons did not significantly improve model fit (no-barrier vs. barrier conditions: χ^2^ = 1.1734, *p* = 0.2787; babble vs. vocoded: χ^2^ = 1.2454, *p* = 0.2644).

**Figure 10:**
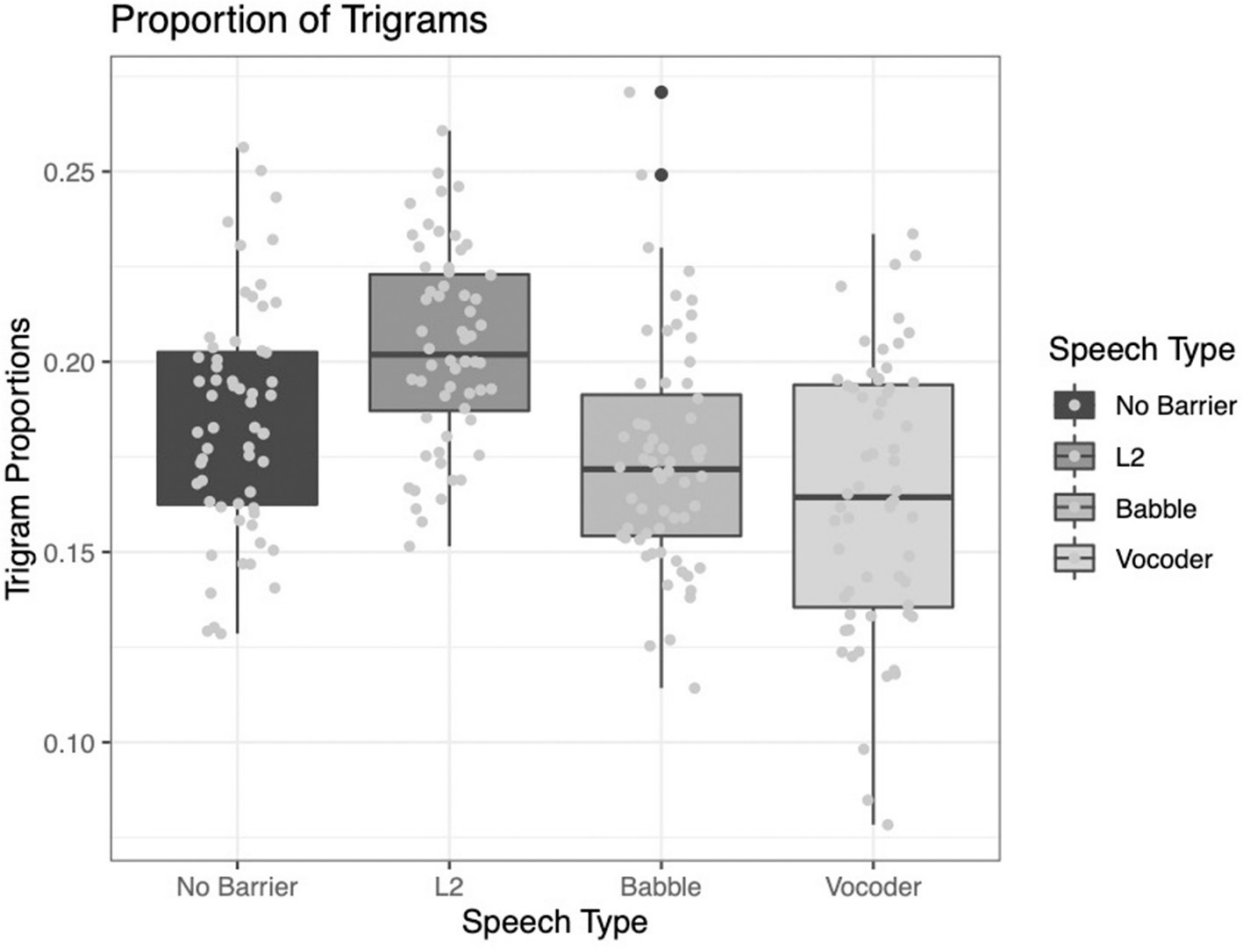
Proportion of trigrams from the LUCID found among the 25,000 most common in the BNC.

Examining Figure 10, it is clear that the L2 condition results in the highest proportion of trigrams among the most frequent in the corpus; however, the other conditions show less clear patterns.

#### Order Effects

As described above, it is possible that condition order, which is conflated with condition itself, may impact the lexical sophistication results. However, as in the case of lexical diversity described in Section Order Effects above, we believe that predicted order effects would be the opposite of the condition effects we see in the present data (i.e., the L2 and babble conditions should have higher measures of lexical sophistication if the task is easier as talkers adapt to the task, topics, and their partner).

Further, examining order of pictures, we primarily see no significant impact on picture order for the metrics described above. There is one exception, however. Picture order is a significant predictor of the proportion of bigrams within the 25,000 most frequent bigrams (χ^2^ = 63.218, *p* < 0.0001). Picture order does not interact with any other factors in the model (i.e., condition). Examining the data, it appears that talkers use a higher proportion of bigrams among the most frequent bigrams in the first picture of each condition and use a smaller proportion in later pictures. We caution over-interpretation of this particular finding as it is not consistent with the null results for the other metrics. However, it is possible that as listeners adapt to the task they do use slightly less frequent collocations as the task progresses. Some acoustic analyses of this data (Lee and Baese-Berk, 2020) have demonstrated that some acoustic properties of the signal (e.g., vowel duration) also decrease across pictures, suggesting that perhaps some aspects of speech do differ as the speaker adapts to speech within a condition^4^.

#### Interim Summary

Overall, the results of lexical sophistication demonstrate similar results to the lexical diversity results above. On average, the no-barrier condition is different from the barrier conditions across many indices, indicating that conditions designed to elicit clear speech not only elicit different numbers of unique words but also different kinds of words. Further, on many metrics the L2 condition differs from the other conditions designed to elicit clear speech. However, the vocoded and babble conditions demonstrate less clear patterns. Indeed, on some metrics they pattern more closely with the no-barrier condition than with the L2 condition, suggesting that different listeners may elicit different types of clear speech. These results are particularly remarkable because the semantic content of the speech is relatively constrained by the pictures being described. That is, talkers do not have unlimited access to use any lexical items they would like. Instead, they are at least somewhat constrained by the task.

## Discussion

Overall, our findings suggest that talkers do, indeed, modulate the lexical diversity and lexical sophistication of their speech as a function of who they are talking to and in what conditions they are producing their speech. Below, we briefly discuss the implications of these findings for our understanding of clear speech, their implications for our understanding of speech processing and communication more broadly, and propose some future directions for investigation.

### Implications for Understanding of Clear Speech

Previous studies of clear speech have largely treated the speaking style as a monolithic construct encompassing all types of scenarios in which a talker might want to produce clearer speech for a listener. Indeed, in studies that have elicited clear speech in the laboratory for a hypothetical listener, the listener is often given a number of options for who they should be envisioning as the recipient of their speech. For example, a common instruction is to “speak as though you are talking to someone who has difficulty hearing or is a non-native speaker of a language” (Picheny et al., 1985; Biersack et al., 2005; Maniwa et al., 2009), which conflates two of the scenarios examined here.

At the same time, clear speech is often described explicitly as a “listener-oriented” speaking style. This is likely largely because the acoustic modifications seen in clear speech correlate with robust improvements in a variety of perceptual measures including objective number of words understood (intelligibility) and subjective difficulty understanding the speech (comprehensibility). However, if this speaking style is truly listener oriented, wouldn't one expect that at least some of the modifications ought to be tailored toward the specific listener one encounters?

Indeed, here we demonstrate that listeners do appear to not only modulate the lexical content of the speech they produce in clear speech conditions, but also modulate this content differently for different types of communication situations. This finding is consistent with previous research suggesting that speakers do alter their speech along different dimensions depending on the identity of the listener. For example, while talkers alter pitch similarly in speech to pets and infants, they only hyperarticulate vowels in IDS (Burnham et al., 2002; Xu et al., 2013). Indeed, other discussions of clear speech research (e.g., Smiljanic and Bradlow, 2011) have suggested the importance of understanding how clear speech might be modulated depending on the audience. While a large body of research has demonstrated that different populations benefit differently from aspects of clear speech (e.g., non-native listeners of differing proficiency levels benefit differently from clear speech), the specific interaction of how talkers specifically modulate their speech (and how listeners may or may not benefit from these modulations) remains understudied.

A skeptical reader may ask whether these results could be due to some factors we are not capturing by comparing across these conditions. However, we believe that the most obvious of these factors are indeed controlled in the current data. One concern, for example, might be that some talkers are more or less likely to modulate their speech for their listener. However, each talker in the corpus used for this analysis appeared in three of the four conditions.

A concern that might be more directly related to the issues of lexical diversity and sophistication investigated here is the influence of topic on these results. That is, if talkers are in truly natural conversations, they can choose the lexical content they produce with relative freedom. Some topics may be more or less likely to elicit more diverse or sophisticated lexical items. One feature that makes this corpus ideal for an analysis like ours is that the semantic content is relatively constrained. For example, one would be relatively surprised to hear a talker discussing nuclear physics when describing the beach scene. This feature, we believe, stacks the cards against us finding the results we did. That is, because the lexical content is relatively constrained, it is even more remarkable to see effects of lexical diversity and sophistication emerge.

We believe that these findings have two important implications for our understanding of clear speech. The first is that typical investigations of clear speech focus on acoustic properties of the speech or on perceptual consequences of clear speech for listeners. Our findings suggest that clear speech encompasses a set of speaking styles that differ from plain speech not only on acoustic dimensions but also on other dimensions, including lexical selection.

The second is that a more nuanced understanding of clear speech is necessary to fully understand the phenomenon (or set of phenomena). That is, while clear speech as an overarching style does, clearly, have some characteristics that are common, it does appear that this speech is listener-oriented in a more specific way. Talkers modify their speech for their listeners (as seen in the differentiation of L2 speech from the other two clear-speech eliciting conditions) and, in some situations, depending on the communication situation with a single listener (i.e., babble vs. vocoded speech). These results open new avenues for exploration, which we describe in more detail in section Future Directions and Open Questions below.

### Audience Design, Speech Production, and Predictability

In some ways, these results are unsurprising. As discussed in the introduction of this paper, it has been clear for decades that talkers modulate their speech for their listener. Indeed, this modulation, often described as “audience design” (Clark and Murphy, 1982) can take many forms including modulating speaking style (Bell, 1997) and modulating referents to given or new items (Horton and Gerrig, 2002). However, a speaker's ability to modulate their speech for specific audiences is impacted by many factors, including memory demands (Horton and Gerrig, 2005). Further, it is not fully clear how audience design may impact lexical selection beyond modulating items within the common ground (Horton and Gerrig, 2002, 2005) or entraining on a shared term to refer to an object (Clark and Wilkes-Gibbs, 1986; Brennan and Clark, 1996; Metzing and Brennan, 2003). That is, it is unclear how much speakers modulate lexical sophistication or lexical diversity as a function of their audience.

Indeed, tracking the frequency of lexical items used is, on its surface, rather complicated. Tracking the frequency and appropriately modulating the frequency of collocations of words appears to be even more complicated. While we do not suggest that speakers are consciously modulating the frequency of words or collocations that they use, it is important to note that speakers do have some metalinguistic awareness of lexical frequency (Carroll, 1971; Verhagen and Mos, 2016). That is, they are aware of what words are relatively higher and lower frequency, suggesting that modulating such factors in their speech may not be as complicated as it initially sounds.

One fundamental question is *why* speakers might modulate their speech in the ways we observe here. We have suggested throughout the paper that this modulation may result in speech that is easier to understand. But easier how, exactly? One way in which the speech may be easier to understand is that it may be more predictable for the listener. It is clear that semantic predictability within a sentence impacts perception. Low predictability sentences (e.g., *mom thinks that it is yellow*) are less well-understood than high-predictability sentences (e.g., *the color of a lemon is yellow*; Kalikow et al., 1977). Similarly, semantically anomalous sentences (e.g., *the black top ran the spring*) are harder to understand than semantically meaningful ones (Miller and Isard, 1963). On a lexical level, high frequency words are perceived more accurately than low frequency words (Carroll, 1971; Verhagen and Mos, 2016). Caregivers use more repetition and a more restricted vocabulary when talking to 6-month-old infants than to 3-month-old infants (Genovese et al., 2020), but a larger and more diverse vocabulary again as infants age and develop more adult-like linguistic abilities (Genovese et al., 2020; Tal et al., 2021). In addition, native talkers, when communicating with non-native talkers, have been found to avoid idiomatic expressions and use more high-frequency words (e.g., Rodriguez-Cuadrado et al., 2018). This suggests that, in both IDS and foreigner-directed speech, talkers make efforts to modulate their lexical choices to avoid confusion, and aid non-native or young listeners through a preference for common words, and phrases that are less semantically ambiguous. Therefore, it could be the case that decreased lexical sophistication results in speech that is slightly more predictable, and thus easier to understand. Another potential argument is supported by claims that talkers may, to an extent, imitate or match certain characteristics or features of infant-speech or foreigner speech when modifying their own speech to aid in communication (Ferguson, 1975). The decrease in lexical diversity and lexical sophistication could be an effort to match the diversity and sophistication of their communicative partner when considering the L2 condition.

Language users modulate their speech in discourse to disambiguate referents as much as possible, which also aids comprehension. Arnold (2008) suggests that modulations in how referents are expressed in discourse are functions of speakers making larger-scale decisions about the level of an addressee's knowledge based on shared social groups or other information that is available about the addressee, and smaller-scale adjustments throughout a conversation depending on the conversation's focus, topic, and whether the information being discussed is given or new. In environments where it is particularly difficult for interlocutors to understand each other, they may resort to different methods of referring to objects in the world than they would in environments where conversation is easier to understand. This would predict increased lexical diversity in the no-barrier condition compared to the other conditions, which is what we observed. These findings potentially support previous literature highlighting the adaptive and instructive nature of foreigner-directed speech, in that talkers seem to modulate their speech in a way that will help with comprehension, and also potentially with acquisition, despite their attitudes toward the speakers themselves (Uther et al., 2007). Thus, given its inherently didactic nature, the trend for lexical diversity to decrease when communicating with non-native talkers may be relatively salient across multiple L2 backgrounds. This trend occurs even though talkers incorporate social information, whether positive or negative, when making judgments about the addressee's prior knowledge.

It is quite clear that the decreased lexical diversity measures also result in more predictable speech. While we have not examined the productions directly, one interpretation of the decreased lexical diversity in the conditions designed to elicit clear speech is that there is an increase in repetition. Previous research on foreigner-directed speech supports this hypothesis by showing that native talkers do tend to employ more repetitions or reduplications in an attempt to help clarify their message (Ferguson, 1975; Rodriguez-Cuadrado et al., 2018). Thus, it is possible that this is what we are seeing through the low lexical diversity scores in the L2 condition. One interesting avenue for future exploration would be whether listeners signal a need for repetition, or whether the speakers choose to provide the repetition without an explicit prompt. It is also possible that clarifications take different forms across conditions. For example, repeating vs. rephrasing may be differently distributed across the conditions. Intuitively, one might expect the L2 condition would result in the most rephrasing, as listeners might be unfamiliar with particular lexical items. However, if our results are due to increased repetition, it appears that we may, in fact, predict the most repetition in those conditions, if we were to directly investigate the conversations in more detail.

Taken together, our results suggest that talkers have extraordinary ability to modify multiple aspects of their speech for their listener. This modulation may impact predictability of speech, making it easier to understand. However, the specific interactions between lexical diversity, sophistication, and predictability in the signal should be investigated in future studies.

### Future Directions and Open Questions

Of course, this project leaves many open questions and avenues for future direction. For example, while we investigate lexical selection in the present study, we do not investigate syntactic or other high-level properties of the language produced by talkers in each condition. One might expect that speakers would demonstrate the most syntactic complexity in the no-barrier condition and the least syntactic complexity in speech to non-native listeners. Similarly, one could investigate “burstiness” (Altmann et al., 2009), or how locally frequent words are. That is, one might expect that in the clear speech conditions talkers may produce more bursty speech, which has more productions of similar words in a short period of time before shifting to a new topic with new lexemes presenting as bursty. In the present study, we only investigate a handful of metrics of the lexical selection by talkers. A number of other lexical properties (e.g., neighborhood density) could provide additional information about the lexical content produced in clear speech and how it might vary across listeners and communication scenarios.

Further, it is important to note that the results of the study are somewhat limited because condition and order of condition are conflated. We do not believe that condition order is the driving factor for our results. If condition order (rather than condition per se) were the source of differences, we would expect to see identical patterns for all metrics in the babble and L2 conditions, which is not what we observe^5^. Additional evidence that condition order alone is driving our results can be found in other work using these same stimuli (e.g., Baker and Hazan, 2011; Lee and Baese-Berk, 2020), which failed to find effects of reduction over the course of a task. That is, Baker and Hazan (2011) fail to find evidence of “learning” across conditions or pictures. Lee and Baese-Berk (2020) find that talkers “re-set” at the start of a new picture in terms of intelligibility of their speech. These findings are consistent with work in the area of second mention reduction which demonstrates that a variety of factors (e.g., topic changes, listener changes, and even narrative devices) can “block” such reductions (acoustic or lexical) from occurring (see, e.g., Fowler et al., 1997). Given these converging results, we do believe that condition, not order, is driving these results. However, future work should counterbalance conditions across orders to ensure that differences we observe are, indeed, driven by condition.

An additional area of inquiry is whether the findings demonstrated here hold throughout a conversation. In some previous work from our lab (Lee and Baese-Berk, 2020), we investigated these same conversations in terms of their acoustic properties and the perceptual consequences. We demonstrated that, in general, speakers produce more intelligible speech when communicating with non-native talkers than native talkers; however, they become less clear over the course of a single conversation. When the topic of conversation switches (i.e., talkers switch to a new picture with the same listener), they “reset” starting over with clearer speech. We interpreted these findings as evidence that what has been previously described as clear speech may have both listener- and speaker-oriented motivations. It is possible that similar patterns of becoming less clear occur with lexical items, though it is less clear whether the “reset” would occur for lexical items shown here.

## Conclusion

In the present study, we investigate speech from naturalistic conversations designed to elicit a clear speaking style. Specifically, we investigate a series of indices of lexical diversity and lexical sophistication in this speech. We find that talkers modulate their speech in terms of both the lexical diversity (i.e., variability of lexical items) and lexical sophistication (i.e., typicality of lexical items). Specifically, talkers show the most lexical diversity and the most lexical sophistication in conversational situations that are designed to elicit plain speech. They demonstrate the least lexical diversity and least lexical sophistication in speech produced for a non-native listener. The results suggest that, in addition to the acoustic modifications previously demonstrated in clear speech work, talkers modulate their lexical selection as well. Further, the results demonstrate that clear speech is not a monolithic construct. Rather, it is a set of speaking styles in which talkers take the listener and communication situation into consideration.

## Data Availability Statement

The datasets generated for this study can be found in online repositories. The names of the repository/repositories and accession number(s) can be found below: https://osf.io/dfhpu/?view_only=49d95d90424941da82217a239ab7450c.

## Ethics Statement

The studies involving human participants were reviewed and approved by University of Oregon, Institutional Review Board. The patients/participants provided their written informed consent to participate in this study.

## Author Contributions

All authors listed have made a substantial, direct and intellectual contribution to the work, and approved it for publication.

## Conflict of Interest

The authors declare that the research was conducted in the absence of any commercial or financial relationships that could be construed as a potential conflict of interest.

## Footnotes

^1^Bradlow, A. R. *SpeechBox*. https://speechbox.linguistics.northwestern.edu.

^2^The sound files for the LUCID corpus and the transcriptions in Praat TextGrids are publicly available in SpeechBox (*https://speechbox.linguistics.northwestern.edu/#!/home*.) Tool for the Automatic Analysis of Lexical Sophistication and Tool for the Automatic Analysis of Lexical Diversity are also both publicly available (*https://www.linguisticanalysistools.org*.) Further, our data (exported from TAALES and TAALED) and code and preregistration for our analyses are available via OSF (*https://osf.io/dfhpu/?view_only=49d95d90424941da82217a239ab7450c*).

^3^Note that this analysis (and any analysis with multiple levels for a single factor in a mixed model) does not allow reporting of a “main effect” of condition, as in a traditional ANOVA (see, e.g., Schad et al., 2020). Therefore, these comparisons are not *post-hoc* comparisons but are the (preregistered) comparisons of interest for this study. At the request of an anonymous reviewer, we also conducted analyses to examine overall effects. The results of these analyses, presented in Supplemental Materials, mirror those reported below. The models for all but one metric have *t*-values >1.85, suggesting a significant difference among conditions. The exception to this is trigram frequency (in Section Trigram Frequency below), which also does not show differences among conditions in our preregistered analysis (reported below in each of the subsections of section Results).

^4^Note, however, that many other aspects of the acoustic signal (e.g., *F*0, speaking rate) and intelligibility do NOT differ as a function of the order of the picture in the task (Lee and Baese-Berk, 2020). Therefore, we reiterate our caution in over-interpretation of this specific result.

^5^It is important to note, however, that given our preregistered analyses, we do not report direct comparisons between the babble and L2 conditions. We believe this is appropriate given that a null result (the predicted result if order effects were significant) would, itself, be difficult to interpret, as it would not provide conclusive evidence *for* the null hypothesis, it would just fail to provide evidence to reject it.
