# Supplementary material for: Lexical Diversity, Lexical Sophistication, and Predictability for Speech in Multiple Listening Conditions
Source: Front Psychol. 2021 Jun 18;12:661415. doi: 10.3389/fpsyg.2021.661415 (PMC8249744; doi:10.3389/fpsyg.2021.661415)
Supplement: Supplementary file 1 [file Table_1.DOCX]

|  | **Type-Token Ratio** | | | **Moving Average Type-Token Ratio** | | | **Hypergeometric Distribution** | | | **Measure of Textual Lexical Diversity** | | |
| --- | --- | --- | --- | --- | --- | --- | --- | --- | --- | --- | --- | --- |
| *Predictors* | *Estimate* | *standardized std. Error* | *t value* | *Estimate* | *standardized std. Error* | *t value* | *Estimate* | *standardized std. Error* | *t value* | *Estimate* | *standardized std. Error* | *t value* |
| Intercept | 0.26 | 0.07 | 70.80 | 0.65 | 0.10 | 176.38 | 0.75 | 0.09 | 313.33 | 32.88 | 0.09 | 52.00 |
| Barrier vs. No Barrier | 0.05 | 0.04 | 13.78 | 0.04 | 0.04 | 14.38 | 0.02 | 0.05 | 9.69 | 6.40 | 0.04 | 12.43 |
| L2 vs. L1 | -0.03 | 0.05 | -8.26 | 0.01 | 0.05 | 2.80 | -0.00 | 0.06 | -0.43 | 1.20 | 0.05 | 2.07 |
| Babble vs Vocoder | 0.01 | 0.05 | 2.49 | 0.01 | 0.05 | 4.55 | 0.01 | 0.06 | 3.05 | 1.41 | 0.05 | 2.87 |

Table A1: Model Summaries for Sections 4.1.1, 4.1.2, 4.1.3, 4.1.4

|  | **Frequency** | | | **Range** | | |
| --- | --- | --- | --- | --- | --- | --- |
| *Predictors* | *Estimate* | *standardized std. Error* | *t value* | *Estimate* | *standardized std. Error* | *t value* |
| Intercept | 8.75 | 0.08 | 145.69 | 74.56 | 0.10 | 321.67 |
| Barrier vs. No Barrier | -0.61 | 0.05 | -8.71 | 0.17 | 0.05 | 0.81 |
| L2 vs. L1 | 0.26 | 0.06 | 3.58 | 1.85 | 0.06 | 8.18 |
| Babble vs Vocoder | -0.11 | 0.06 | -1.84 | -0.54 | 0.06 | -2.81 |

Table A2: Model Summaries for Sections 4.2.1 and 4.2.2

|  | **Bigram Frequency** | | | **Trigram Frequency** | | | **Proportion Bigrams within 25,000 Most Frequent** | | | **Proportion Trigrams within 25,000 Most Frequent** | | |
| --- | --- | --- | --- | --- | --- | --- | --- | --- | --- | --- | --- | --- |
| *Predictors* | *Estimate* | *standardized std. Error* | *t value* | *Estimate* | *standardized std. Error* | *t value* | *Estimate* | *standardized std. Error* | *t value* | *Estimate* | *standardized std. Error* | *t value* |
| Intercept | 0.33 | 0.09 | 77.20 | 0.02 | 0.12 | 34.48 | 0.58 | 0.09 | 149.20 | 0.18 | 0.09 | 60.97 |
| Barrier vs. No Barrier | -0.02 | 0.06 | -5.23 | 0.00 | 0.06 | 1.65 | -0.02 | 0.05 | -3.75 | 0.00 | 0.05 | 1.09 |
| L2 vs. L1 | 0.00 | 0.07 | 0.75 | -0.00 | 0.07 | -1.35 | 0.04 | 0.06 | 8.00 | 0.02 | 0.06 | 7.30 |
| Babble vs Vocoder | -0.00 | 0.06 | -0.44 | 0.00 | 0.07 | 1.41 | -0.01 | 0.06 | -1.73 | -0.00 | 0.06 | -1.10 |

Table A3: Model Summaries for Sections 4.2.3, 4.2.4, 4.2.5, and 4.2.6

|  | **Type-Token Ratio** | | | **Moving Average Type-Token Ratio** | | | **Hypergeometric Distribution** | | | **Measure of Textual Lexical Diversity** | | |
| --- | --- | --- | --- | --- | --- | --- | --- | --- | --- | --- | --- | --- |
| *Predictors* | *Estimate* | *standardized std. Error* | *t value* | *Estimate* | *standardized std. Error* | *t value* | *Estimate* | *standardized std. Error* | *t value* | *Estimate* | *standardized std. Error* | *t value* |
| Intercept | 0.28 | 0.10 | 42.78 | 0.67 | 0.12 | 130.62 | 0.75 | 0.10 | 223.83 | 36.06 | 0.11 | 42.64 |
| Condition | -0.01 | 0.06 | -3.87 | -0.01 | 0.05 | -9.45 | -0.01 | 0.05 | -5.60 | -2.55 | 0.05 | -8.94 |

Table A4: Model Summaries “overall effects” for Sections 4.1.1, 4.1.2, 4.1.3, 4.1.4

|  | **Frequency** | | | **Range** | | |
| --- | --- | --- | --- | --- | --- | --- |
| *Predictors* | *Estimate* | *standardized std. Error* | *t value* | *Estimate* | *standardized std. Error* | *t value* |
| Intercept | 8.56 | 0.09 | 94.80 | 75.68 | 0.10 | 257.21 |
| Condition | 0.15 | 0.06 | 3.72 | -0.68 | 0.05 | -6.01 |

Table A5: Model Summaries for Sections 4.2.1 and 4.2.2

|  | **Bigram Frequency** | | | **Trigram Frequency** | | | **Proportion Bigrams within 25,000 Most Frequent** | | | **Proportion Trigrams within 25,000 Most Frequent** | | |
| --- | --- | --- | --- | --- | --- | --- | --- | --- | --- | --- | --- | --- |
| *Predictors* | *Estimate* | *standardized std. Error* | *t value* | *Estimate* | *standardized std. Error* | *t value* | *Estimate* | *standardized std. Error* | *t value* | *Estimate* | *standardized std. Error* | *t value* |
| Intercept | 0.32 | 0.10 | 54.85 | 0.02 | 0.11 | 30.27 | 0.59 | 0.10 | 103.10 | 0.20 | 0.09 | 48.86 |
| Condition | 0.01 | 0.06 | 3.53 | 0.00 | 0.06 | 0.09 | -0.00 | 0.06 | -1.84 | -0.01 | 0.06 | -5.06 |

Table A6: Model Summaries for Sections 4.2.3, 4.2.4, 4.2.5, and 4.2.6
